# Dr. Crichton's Case of Oedema Fugax

**Published:** 1801-07

**Authors:** A. Crichton

**Affiliations:** Clifford Street


### To Dr. BRADLEY.

My dear Sir,

I Have fent you two papers of which I lately fpoke to you. The firft relates to a difeafe which I do not confider as very uncommon, yet which is not defcribed in any Syftem of Nofology or Practice of Phyfic which I am acquainted with. It is a variety of œdema, which differs in a great number of circumftances from all other œdematous fwellings, and which, on account of the fuddennefs of its appearance and departure, and the frequency of its attack in a fhort fpace of time, I have prefumed to call *Oedema Fugax*.

The other cafe is much rarer, and is equally undefcribed. If you deem thefe papers worthy of being made public, you are at perfect liberty to infert them in your Journal. I am,

Dear Sir,

Your's very fincerely,

*Clifford Street, June* 10, 1801. A. CRICHTON.

### I. *Case of Oedema Fugax.*

A widow lady, upwards of 60 years old, of a good healthy conſtitution, which had not been injured by any intemperance or irregularity of life, was ſuddenly ſeized one evening, about a month ago, with a confuſed pain in her head, giddineſs, and general uneaſineſs; ſoon after which her face and the back of her hands began to ſwell, and increaſed to a prodigious ſize. She became feveriſh, according to her own deſcription, and paſſed the whole evening and the greater part of the night in pain and anxiety. Her eye-lids were ſo ſwollen, ſhe ſaid, that ſhe could ſcarcely ſee. Towards morning ſhe became better, fell into a ſleep, and awoke almoſt entirely free from all the above-mentioned ſymptoms; but ſhe felt great weakneſs, and a total loſs of appetite. Her face and hands ſtill remained a little ſwelled, but in ſo trifling a degree, in compariſon with what had happened the night before, that ſhe paid no more attention to them; and imagining the whole to proceed from indigeſtion, ſhe took, of her own accord, a domeſtic purge, which ſhe had occaſionally taken to obviate coſtiveneſs. The medicine operated well, but did not improve her appetite, which ſeemed quite gone. In the afternoon ſhe again began to grow uneaſy, and to ſuffer from head-ach, and again her face and hands ſwelled greatly. She paſſed a bad night, and ſent for me early the next morning.

When I ſaw her, which was about noon, ſhe had no other remains of the ſwelling than a little puffineſs under each eye, and on the back of the hands. Her tongue was foul, but not greatly ſo; her ſkin was of a natural degree of heat; the pulſe beat about 96 in a minute, and was full and hard, but the hardneſs of the pulſe did not ariſe ſo much from a quick and full diſtenſion of the artery as from that rigidity of the coats of the artery which is natural to old age.

I was informed that there was not any thing uncommon either in the quantity or quality of the urine ſhe paſſed, or in her ſtools; ſhe had not the ſmalleſt appetite. The lighteſt ſoup, if it taſted of meat, diſguſted her, yet ſhe had not any nauſea, or any bad taſte in her mouth.

As her bowels were perfectly open, and had been thoroughly evacuated, according to report, by the medicine ſhe had taken the day before, and as ſhe complained much of a ſenſe of general weakneſs, I ordered her the following tonic and gentle aperient combined:

℞. Pulv. rad. columb. gr. viij. Aq. cinnamon ℥ij. Aq. diſtillat. ℥x. Tinct. rhæi. Tinct. card. comp. aa. ℥ſs. M. ft. hauſtus 6ta. q. hora ſumendus.

When

When I ſaw her next day, ſhe informed me that ſhe had had a ſevere return of her complaint, and had paſſed a very reſtleſs night. She felt a little ſtronger, however, but her appetite was as bad as it had been the day before: Her face and hands were ſwelled when I viſited her, but only ſlightly: The ſkin was colourleſs, but had a gloſſy hue, and appeared to be diſtended by a ſerous fluid. When the finger was preſſed on the tumefied parts, it left an impreſſion only for a ſhort time, and by no means ſo diſtinct as in anaſarca: She had had two ſtools, which were of a healthy aſpect. Her tongue was ſtill fouler than it had been the day before, yet ſhe had not any diſagreeable taſte in her mouth, or great thirſt, or head-ach; but as theſe ſymptoms had occurred in the beginning of her complaint, and as the colombo did not agree with her, I ſuſpected her ſtomach might be loaded with offenſive matter, and accordingly I ordered her an emetic, conſiſting of fifteen grains of ipecac. and one grain of tartar emetic, in a draught. I likewiſe ordered her ſix grains of calomel, in a pill, at bed time.

I was informed the next day that the emetic had operated rather powerfully, but that there was not any thing uncommon in the appearance of what ſhe had vomited. It conſiſted almoſt entirely of the fluid ſhe had drank, and a little mucus from the coats of the ſtomach: The ſwelling had occurred both in the middle of the day and towards evening. Her appetite was as bad as ever, and ſhe now complained of great weakneſs.

In the courſe of my practice, I had met with a few caſes which were nearly ſimilar to this, in which I had puſhed tonics in the beginning, both alone and combined with diuretics, and alſo with gentle purges; but in all which caſes, I thought they had done harm. Still I was tempted to have recourſe to them again in this caſe, and I ordered my patient two ſcruples of cream of tartar, with a few grains of ginger, morning, noon, and night, with a view of acting on the kidneys, and deſired her to take two ounces of the decoction of yellow bark, with a drachm of the ſpt. juniperi, after each doſe of the powders.

Theſe did no good: She was in every reſpect the ſame next day as ſhe had been the day before, except that ſhe was weaker. She had voided more urine than uſual, yet the ſwellings returned in as great a degree as before: Her tongue was ſtill of the ſame dirtyiſh yellow colour, and ſhe had not the ſmalleſt appetite. There were ſtill two reſources which I had my choice of in ſuch a caſe: the one was to lay aſide all medicine, and give my patient as much light food as poſſible, and truſt to Nature; the other was to give wine and mercury in

 moderate moderate dofes, which medicines I fufpected, from fome diftant analogies which had prefented themfelves to me, might be ufeful. I preferred the laft, for the fake of inftruction, and accordingly ordered my patient a glafs of port wine every four hours, and five grains of the pilula hydrargyri every night. I did not fee her again until the fecond day afterwards, when I was moft agreeably furprized by her telling me, that fhe found her appetite return after the third dofe of her wine, and that fhe had paffed a tolerably good night, but ftill had fome return of the fwelling. The next day fhe was ftill better, and her appetite was become keen; her tongue was clean, her pulfe good, and there were fcarcely any remains of the fwelling. In a few days fhe was quite well.

Of this kind of œdema, the cafes which I have met with, have either yielded very eafily to evacuations and gentle tonics, or proved very obftinate, and refifted almoft every medicine. In one cafe which fucceeded an intermittent fever, which had been very much neglected for many months, and had been badly managed afterwards, an hectic fever occurred, which proved fatal. I was furprized to obferve how fuddenly the fwelling occurred in this cafe, and to what a degree it fometimes arofe. When it occupied the face, the patient was completely disfigured, and the palpebrae became fo much diftended, as to clofe the eyes entirely. Sometimes the feet and legs only were fwelled; and this did not depend on any particular fituation, but occurred both when the patient was in bed, and when fitting up, fo that in this refpect it differed very much from common anafarca.

Not only the immediate or proximate caufes of the fwelling in this difeafe are involved in great obfcurity, but the exciting caufes alfo are very obfcure: Whatever the immediate caufes may be, it is evident that their action is very tranfitory. Any fwelling containing a fluid, or gelatinous exfudation, may arife on the one hand, either from a morbidly increafed or diminifhed action of thofe ramifications of the arterial fyftem which fecrete colourlefs fluids, or on the other hand, from a torpor, weaknefs, or kind of palfy in the abforbents. This laft caufe, however, only determines the fwelling, but not the nature of the fluid effufed. In the abovementioned cafes, the fluid appeared to be gelatinous, and we are therefore induced to believe, that the fecreting veffels were as much in fault as the abforbents, for it is not a gelatinous fluid which is ufually fecreted in the cavities of the cellular fubftance. This fuppofition, however, affumes a pofition as being granted, which is by no means well made out: I mean, that moft of the altered appearances, or qualities of fecreted fluids, depend pend on the action of the veffels which fecrete them. This fuppofition is doubtlefs fupported by a multitude of facts; but there are alfo many facts which ftand in oppofition to it, and incline us to believe, that there are chemical changes occurring in every part of our frame, independently of all vital actions, and which have a great influence on the fecretions of every kind. In a difeafed ftate thefe are frequently multiplied, and new compounds are formed which produce the moft fingular phenomena, fuch as eruptions, hæmorrhagies, fwellings, and fpafms; but the nature of thefe chemical changes are almoft entirely unknown to us; and if they were known, it is extremely doubtful how far they would ferve us as ufeful guides in practice. In the above-mentioned cafe I directed my efforts at laft to the altering the general action of the fanguiferous and abforbent fyftem rather than to the correcting any particular chemical acrimony.

### II. *Cases of an hitherto undescribed Cause of Jaundice.*

The jaundice which I am about to defcribe is unfortunately always a fatal one in its advanced ftages; and what adds to the misfortune of fuch a circumftance is, that it cannot be diftinguifhed in its commencement, as far as I know, from the more favorable fpecies of icterus. Although, therefore, I have not the fatisfaction to fuppofe that I fhall contribute any thing to the general good of mankind by this communication, yet I hope to render a fervice to practitioners, by pointing out a fymptom which will warn them of the truly defperate fituation of their patient, and enable them to foretell to the patient's friends, in fufficient time, the melancholy nature of the cafe.

The firft inftance of this kind of jaundice which I met with, was that of an old woman who was admitted into the Weftminfter Hofpital nearly five years ago.

The whole of her fkin and the tunica albuginea of the eyes were of a deep orange colour, her urine was alfo of a deep orange colour, and depofited a thick dark-coloured fediment on cooling; fhe had great languor and fenfe of weaknefs, but fhe had fewer dyfpeptic fymptoms than are commonly met with in jaundice. Her appetite was good, her tongue was quite clean, fhe had no bitter tafte in her mouth, nor had fhe any head-ach, but fhe complained much of thirft, and of a fevere purging with which fhe had been fubject two or three days before fhe applied for admiffion into the houfe. This patient had never had any pain in her right fide, neither was fhe affected with cardialgia, naufea, or much flatulency. Her pulfe was of the natural quicknefs, but was fmall and feeble.

Her complaint began about a fortnight before fhe came to the the hofpital. It was fo imperceptible in its progrefs, and attended with fo little uneafinefs of any kind, that fhe had not applied before for any advice.

As the diarrhœa was evidently of a critical kind, and fhe was finking under it, it was the firft object of my care. I ordered her a gentle cordial with four drops of laudanum to be taken every four hours, and gave orders that fhe fhould be frequently fupplied with light mild nourifhment.

The diarrhœa was at firft ftopt by the laudanum, but began to make its appearance again in about fourteen hours, and in thirty hours was as bad as at the beginning. I then ordered her to take the confect. opiata with the cretaceous mixture every four hours. This at firft checked the diarrhœa, but it again began to return in ten or twelve hours; and although I had left orders to increafe the quantity of opium, yet it was not checked, but returned at intervals, and in two days was as bad as ever, although at that time the patient took nearly one grain of opium every four hours. I now had recourfe to other aftringents, particularly the extractum ligni campechenfis, and ext. catechu, but thefe did not check the diarrhœa in any degree; it increafed notwithftanding every effort was made to ftop it, and the patient died quite exhaufted on the fixth day after her admiffion into the hofpital. To the laft moment her ftools continued of a pale clay colour. Her pulfe never varied in quicknefs until near her death, when it fuddenly rofe to about a hundred in a minute. The colour of the fkin and of her urine became of a more and more intenfe yellow colour during the whole period of her complaint.

Upon infpecting the body, the caufe of the difeafe foon prefented itfelf. There were no gall ftones either in the gall bladder or in the ductus communis choledochus, but the gall bladder and its duct, and the ductus hepaticus, were diftended with bile to a preternaturally large fize. It was not poffible to force any of the bile into the duodenum; and upon feeking for the caufe of this obftruction, we foon found that a portion of the ductus communis was furrounded by the extremity of the pancreas, which was completely in a fcirrhous or indurated ftate. The duct was fo much compreffed that it was impoffible to pafs the fmalleft filver probe through it without tearing it.

Another cafe fimilar to this prefented itfelf to me about a year afterwards in another old woman, who alfo was admitted a patient of the Weftminfter hofpital. The difeafe was completely formed before fhe applied for relief. She had no pain in her fide, but was troubled with flatulency. A diarrhœa had come on a day or two before her admiffion into the houfe, and what fhe voided in this way fhe faid was very pale. As foon as I afcertained the truth of this, I communicated to the pupils who were prefent my fufpicions of the cafe being a fatal one, and told them what I fufpected to be the caufe of the complaint. I ordered a large dofe of ftrong mercurial ointment to be rubbed in every night and morning, and gave her aftringents combined with cicuta, but the relief fhe obtained was as temporary as in the firft cafe, and fhe died in a few days quite exhaufted. The body was opened after death, and the very fame appearances difcovered.

Since that time I have only met with one cafe in private practice which I confidered as fimilar to thefe. It occurred in a gentleman of about fifty years of age, who had enjoyed tolerably good health until he was attacked with this complaint. I am forry to fay I could not verify my fufpicions as to the real nature of the cafe, inafmuch as I was denied the permiffion of examining the body.

Melancholy as thefe cafes were in regard to their event, they afforded me a certain degree of fatisfaction, both by teaching me a new fact in the hiftory of jaundice, and by correcting an error which I had long entertained, and which I have reafon to think is very common: I mean, that a great number of the anomalous fymptoms of dyfpepfia, particularly fpafms of the ftomach, are often fymptomatic of difeafed pancreas. Here are three cafes, two of which certainly ftand in oppofition to this fentiment, and feem to prove that the ftomach does not, at leaft in all cafes, fympathize with a difeafed ftate of pancreas, even with fuch a difeafed ftate as muft deftroy its functions.

The above cafes will, I truft, be fatisfactory to young practitioners, who may not have feen fuch cafes, as they will be taught by them to be very guarded in their prognofis, when they meet with fuch a diarrhœa in jaundice as I have defcribed. And it gives me pleafure to fend you this account, fince I am perfuaded you muft recollect being prefent at the infpection of the bodies of the two women.

